# TNFα Modulates Fibroblast Growth Factor Receptor 2 Gene Expression through the pRB/E2F1 Pathway: Identification of a Non-Canonical E2F Binding Motif

**DOI:** 10.1371/journal.pone.0061491

**Published:** 2013-04-16

**Authors:** Sirio D’Amici, Simona Ceccarelli, Enrica Vescarelli, Ferdinando Romano, Luigi Frati, Cinzia Marchese, Antonio Angeloni

**Affiliations:** 1 Department of Experimental Medicine, Sapienza University of Rome, Rome, Italy; 2 Department of Public Health and Infectious Diseases, Sapienza University of Rome, Rome, Italy; 3 Department of Molecular Medicine, Sapienza University of Rome, Rome, Italy; Northwestern University, United States of America

## Abstract

Interactions between epithelium and mesenchyme during wound healing are not fully understood, but Fibroblast Growth Factors (FGFs) and their receptors FGFRs are recognized as key elements. FGFR2 gene encodes for two splicing transcript variants, FGFR2-IIIb or Keratinocyte Growth Factor Receptor (KGFR) and FGFR2-IIIc, which differ for tissue localization and ligand specificity. Proinflammatory cytokines play an essential role in the regulation of epithelial-mesenchymal interactions, and have been indicated to stimulate FGFs production. Here we demonstrated that upregulation of FGFR2 mRNA and protein expression is induced by the proinflammatory cytokines Tumor Necrosis Factor-α, Interleukin-1β and Interleukin 2. Furthermore, we found that TNFα determines FGFR2 transcriptional induction through activation of pRb, mediated by Raf and/or p38 pathways, and subsequent release of the transcription factor E2F1. Experiments based on FGFR2 promoter serial deletions and site-directed mutagenesis allowed us to identify a minimal responsive element that retains the capacity to be activated by E2F1. Computational analysis indicated that this element is a non-canonical E2F responsive motif. Thus far, the molecular mechanisms of FGFR2 upregulation during wound healing or in pathological events are not known. Our data suggest that FGFR2 expression can be modulated by local recruitment of inflammatory cytokines. Furthermore, since alterations in FGFR2 expression have been linked to the pathogenesis of certain human cancers, these findings could also provide elements for diagnosis and potential targets for novel therapeutic approaches.

## Introduction

Interactions between epithelium and mesenchyme play a vital role in the control of growth and differentiation of epithelium during embryonic development, morphogenesis and wound healing of several tissues [1]–[3]. When disturbed, such interactions may also result in pathological states [4]–[6]. The nature of the mesenchymally derived signals responsible for epithelial growth and differentiation has been extensively investigated. These epithelial-mesenchymal interactions can be mediated by extracellular matrix components, cell surface-associated molecules or soluble growth factors, such as cytokines. The latest are major characters involved in such signals, as they can act in an autocrine and/or paracrine fashion without requirement of cell-cell contact. However, the three types of signals are not mutually exclusive because the action of one may be dependent on or mediated by the expression of others [7].

Among the cell membrane-associated molecules, Fibroblast Growth Factor Receptors (FGFRs) act as important modulators of mesenchymal-epithelial interactions, being involved in many biological processes during embryo development and homeostasis of adult body tissues. The FGFRs gene family consists of four highly related genes, FGFR1 to 4, encoding polypeptides that are 55% to 72% identical in their amino acid sequence. FGFR1 and FGFR2 exhibit broad but distinct patterns of expression during development and in adult tissues, while FGFR3 and FGFR4 have more restricted expression patterns [8].

In particular, FGFR2 gene encodes for two splicing transcript variants, the Keratinocyte Growth Factor Receptor (KGFR or FGFR2-IIIb) and the FGFR2-IIIc. The FGFR2-IIIc, as well as its ligands, is expressed in cells of mesenchymal lineage, while KGFR is predominantly expressed by epithelial cells and its specific ligands, namely Keratinocyte Growth Factor (KGF/FGF7), FGF10 and FGF22, are expressed exclusively by fibroblasts [9]. Such paracrine way of action makes KGFR a good candidate for a key role in the regulation of epithelial-mesenchymal interactions during both physiological and pathological processes.

Among the soluble factors involved in dermal-epidermal interplay, a number of cytokines such as interleukin (IL)1β, IL2, IL6, interferon γ (IFNγ) and tumor necrosis factor α (TNFα) have been primarily interpreted as mediators of inflammatory and/or immunomodulatory reactions [10]. They exert their functional role in the regulation of tissue repair and homeostasis by inducing the expression of other proinflammatory mediators from many cell types, by stimulating keratinocyte migration and proliferation and leukocyte recruitment in cutaneous wounds, by enhancing the production of matrix metalloproteinases by fibroblasts [11]–[14]. Such activities are performed through a dynamic and reciprocal modulation with growth factors expression in keratinocytes and fibroblasts [15]–[16].

One of the most important physiological processes involving both keratinocytes and fibroblasts is wound healing. In fact, an inflammatory response is elicited at the wounded site, which contributes to the modulation of migration, proliferation and differentiation of epithelial and mesenchymal cells, giving rise to the formation of new tissue and ultimately wound closure. This process is regulated by a complex signaling network involving numerous growth factors, cytokines and chemokines [17]. The key role of KGF in wound healing is demonstrated by its increased expression during re-epithelialization of normal human skin. Elevated KGF transcript levels have been also reported in a mouse wound healing model [14]. Previous studies demonstrated that KGF expression is stimulated by IL1β and TNFα [18]–[21]. At the same manner, the release of other growth factors involved in wound healing, like basic FGF and vascular endothelial growth factor (VEGF), from endothelial cells is induced by the presence of proinflammatory cytokines, such as TNFα, IL1β and IFNγ [22]–[23]. More recently, some of these cytokines have been postulated to regulate KGFR [24], whose expression is strikingly modulated during wound repair [25]. In particular, signaling through KGFR is critical for normal wound healing, as demonstrated by the delayed wound re-epithelialization in transgenic mice expressing a dominant-negative KGFR mutant in basal keratinocytes [26].

Moreover, our previous works have identified an increased expression of both KGFR and FGFR2-IIIc also in pathological events, such as the progression and stadiation of classic Kaposi Sarcoma and dermatofibroma, two tumors involving the dermal compartment characterized by a prevalent inflammatory component [27]–[28] and elevated levels of proinflammatory cytokines [22], [29].

Although a large number of factors taking part in wound healing are now well recognized, the events underlying this process need in-depth study.

To identify the mechanisms of KGFR involvement in epithelial repair and homeostasis, we decided to investigate the potential activity of cytokines in controlling the expression of FGFR2 gene in both epithelial and mesenchymal cells.

Furthermore, the genetic analysis of many promoters has significantly contributed to a better understanding of differential gene expression. To date the promoter regions of all murine FGFRs have been identified and characterized using deletion constructs and sequence analysis [30]–[33], however the mechanisms of their transcriptional regulation are still not well understood.

The transcription factors belonging to the E2F family are key participants in a number of cellular events, such as the control of cell cycle, DNA synthesis and gene transcription [34]. In mammals, eight members of the E2F family have been identified and divided into four homology groups: E2F1 to 3, E2F4 and 5, E2F6, E2F7 and 8 [35]. The transcriptional activity of E2Fs is negatively regulated by the product of the retinoblastoma tumor suppressor gene (pRb), or by the related family members p107 and p130 [36]. Previous works found that the increase in expression of human FGFR1 and murine FGFR2 could be mediated through the activation of the pRb/E2F pathway [37]–[39]. Furthermore, it has been demonstrated that transcription of mouse FGFR2 gene is directly activated by E2F1 and suppressed by pRb [37].

In this study, we investigated the cytokine-induced modulation of human FGFR2 gene expression, trying to identify potential factors responsible for this phenomenon. Furthermore, we assessed the role of the transcription factor E2F1 on human FGFR2 promoter activation and characterized E2F1 minimal responsive element through site-directed mutagenesis.

## Materials and Methods

### Ethics Statement

All experiments with human fibroblasts cultures were approved by the Ethics Committee of the Azienda Policlinico Umberto I of Rome (official name of the committee). Written informed consent was obtained from all donors before entering the study. No patients information was shared with the researchers.

### Reagents and Cell Cultures

The human breast adenocarcinoma cell line MCF-7 and the human embryonic kidney HEK293 cells were obtained from the American Type Culture Collection (ATCC-LGC Promochem, Teddington, UK) and cultured in Dulbecco’s Modified Eagle’s Medium (DMEM; Invitrogen, Karlsruhe, Germany), supplemented with 10% fetal bovine serum (FBS; Invitrogen) and antibiotics. Human primary fibroblasts were obtained from a healthy donor skin biopsy of about 1 cm^2^, as previously described [40], and maintained in DMEM containing 10% FBS. The skin biopsies were collected at the Department of Plastic and Reconstructive Surgery of the Azienda Policlinico Umberto I of Rome, as a part of routine treatment, and then transferred to our laboratory for processing.

Recombinant human IL1β and IL2 were purchased from Peprotech Inc. (Rocky Hill, NJ, USA), while IL6, TNFα and IFNγ were from Invitrogen. Human recombinant KGF was purchased from Upstate Biotechnology (Lake Placid, NY, USA). The p38 inhibitor SB202190 was purchased from Sigma-Aldrich srl (Milano, Italy) and used at 10 µM. The C-Raf inhibitor GW5074 was purchased from Santa Cruz Biotechnology (Santa Cruz, CA, USA) and used at 1 µM.

### Immunofluorescence Microscopy

Cells, grown on coverslips, were serum starved for 16 h and then treated for 24, 48 or 72 h with 10 ng/ml IL1β, IL2, IL6, TNFα or IFNγ, and with 10 ng/ml human recombinant KGF as a positive control. Cells were fixed in 4% paraformaldehyde in phosphate-buffered saline (PBS) for 30 min at 25°C, followed by treatment with 0.1 M glycine in PBS for 20 min at 25°C and with 0.1% Triton X-100 in PBS for additional 5 min at 25°C to allow permeabilization. To assess proliferation, cells were incubated with an anti-Ki67 rabbit polyclonal antibody (1∶50 in PBS; Zymed Laboratories, San Francisco, CA, USA), which identifies cycling cells. The primary antibody was visualized using Texas Red conjugated goat anti-rabbit IgG (1∶100 in PBS; Jackson ImmunoResearch Laboratories, West Grove, PA, USA). Nuclei were visualized using 4′, 6-diamido-2-phenylindole dihydrochloride (DAPI) (1∶10000 in PBS; Sigma-Aldrich). Fluorescence signals were analyzed by recording stained images using a cooled CCD color digital camera SPOT-2 (Diagnostic Instruments Incorporated, Sterling Heights, MI, USA) and Axiovision software (Carl Zeiss Inc., Oberkochen, Germany). The percentage of Ki67-positive cells was evaluated by counting, for each treatment, a total of 500 cells, randomly taken from ten microscopic fields in three different experiments, expressed as mean value ± standard deviation (SD) and reported as a graph.

### Immunoprecipitation and Western Blot Analysis

For Western blot analysis, MCF-7 cells untreated or treated with different doses (10 or 100 ng/ml) of cytokines for different times (1, 3, 8, 24, 48 or 72 h) were lysed in RIPA buffer. Total proteins (50–150 µg) were resolved under reducing conditions by 7% SDS–PAGE and transferred to Immobilon-FL membranes (Millipore, Billerica, MA, USA). For KGFR detection, the membranes were incubated overnight at 4°C with anti-Bek, a rabbit polyclonal antibody raised against the intracellular domain of KGFR/FGFR2 (C-17; 1∶200 dilution; Santa Cruz), followed by a goat anti-rabbit horseradish peroxidase (HRP)-conjugated secondary antibody (Sigma-Aldrich). Bound antibody was detected by enhanced chemiluminescence detection reagents (Pierce Biotechnology Inc, Rockford, IL, USA), according to manufacturer’s instructions. For E2F1 detection, the membranes were incubated overnight at 4°C with anti-E2F1 polyclonal antibody (Santa Cruz) diluted 1∶500 for 1 h at 25°C followed by goat anti-mouse-HRP secondary antibody. To assess pRb, p38 and C-Raf phosphorylation, the same membranes were incubated with anti-phospho-pRb, anti phospho-p38 or anti-phospho-C-Raf antibody (Cell Signaling Technology, Inc., Danvers, MA, USA) followed by the appropriate HRP-conjugated secondary antibody. To estimate the protein equal loading, the membranes were rehydrated through washing in Tris buffered saline, stripped with 100 mM β-mercaptoethanol and 2% SDS for 30 min at 55°C and reprobed with anti-Tubulin (1∶1000 dilution; Sigma-Aldrich), anti-p38 or anti-C-Raf antibody (1∶1000 dilution, Cell Signaling Technology). To verify the association between pRb and E2F1, 1 mg of total protein was immunoprecipitated with 4 µg/ml anti-pRb monoclonal antibody. Immunocomplexes, aggregated with 50 µl of γ-bind protein-G sepharose (Amersham Biosciences, Uppsala, Sweden), were washed four times with 0.6 ml of buffer, resolved under reducing conditions by 10% SDS–PAGE and transferred to membranes. Membranes were incubated with anti-E2F1 polyclonal antibody diluted 1∶500 for 1 h at 25°C followed by goat anti-mouse-HRP secondary antibody and enhanced chemiluminescence detection. To estimate the protein equal loading, the membranes were rehydrated, stripped and reprobed with anti-pRb antibodies diluted 1∶1000.

Densitometric analysis was performed using Quantity One Program (Bio-Rad Laboratories srl, Segrate, MI, Italy). Briefly, the signal intensity for each band was calculated and the background subtracted from experimental values. The resulting values were then normalized and reported as relative expression with respect to the control value.

### Quantitative Real-time PCR

Cells, treated as for Western blot analysis, were harvested and total RNA was extracted with the use of TRIzol reagent (Invitrogen). cDNA was generated with oligo(dT) from 1 µg of RNA using the SuperScript III Reverse Transcriptase Kit (Invitrogen). 25 ng of synthesized cDNA was then used for amplification of human KGF using the real-time TaqMan gene expression assay kit (Applied Biosystems by Life Technologies, Carlsbad, CA, USA). For KGFR and FGFR2-IIIc, specific custom TaqMan® Primer/Probe assays were developed (see Table 1) and used at a concentration of 1x per well. A total of 2 µl/well of template was added to the sample wells along with Taqman Universal PCR master mix at a concentration of 1x and water to a volume of 25 µl/well.

**Table 1:** Custom TaqMan Assay gene-specific primers and reporter probes.

GENE	FORWARD PRIMER SEQUENCE	REVERSE PRIMER SEQUENCE	REPORTER SEQUENCE
FGFR2-IIIb	GGCTCTGTTCAATGTGACCGA	GTTGGCCTGCCCTATATAATTGGA	TTCCCCAGCATCCGCC
FGFR2-IIIc	CACGGACAAAGAGATTGAGGTTCT	CCGCCAAGCACGTATATTCC	CCAGCGTCCTCAAAAG

Assays were conducted in triplicate on an ABI 7500 Real Time instrument (Applied Biosystems) using the following conditions: 50°C for 2 min, 95°C for 10 min, and then 95°C for 15 s and 60°C for 1 min, repeated 40 times. Relative quantification was performed using GAPDH mRNA as an endogenous control: for each examined sample, KGFR, KGF or FGFR2-IIIc mRNA expression data were normalized to the GAPDH expression.

### Plasmids Construction

The putative promoter region of the human FGFR2 gene (−1103 to +459 relative to the transcriptional initiation site) and the upstream promoter region (−2235 to −909) were amplified from human genomic DNA by PCR using Prime STAR® HS DNA Polymerase (Takara Bio Inc., Otsu, Japan), inserted into the vector pJet1.2/blunt (Fermentas Inc., Glen Burnie, MD, USA) and then transferred to the final luciferase reporter vector pGL3-basic (Promega, Madison, WI, USA). The primer sequences used are reported in Table 2.

**Table 2:** Primers used for FGFR2 promoter constructs.

CONSTRUCT	FORWARD PRIMER SEQUENCE	REVERSE PRIMER SEQUENCE
−1103 to +459	CTTCATCTATCTTCAGGCCTC	GATGGAGAAAGCGACGAG
−2235 to −909	GCTTATTTACCTATTCACACTCCG	CCTTTTCACTAAGCCGTGTCT
−565 to +459	GGGCAGATGAAATAGAATCAC	GATGGAGAAAGCGACGGAG
−143 to +459	GTGTCTCCGGCTGCTCG	GATGGAGAAAGCGACGGAG
−81 to +114	GGGGGTACCGCGCTGATTGGCAGAGAG	GGGGCTAGCCCGAGCTTTGTGGCGGCCGC
−81 to +58	GGGGGTACCGCGCTGATTGGCAGAGAG	GGGGCTAGCCCGCTCGGCTCTCCACC
−81 to +5	GGGGGTACCGCGCTGATTGGCAGAGAG	GGGGCTAGCCGCCCCCGCCTCCTCGCG

The putative E2F and STAT binding sequences in the 1.5 kb human FGFR2 promoter region from −1103 to +459 and in the 1.3 kb region from −2235 to −909 were searched by means of a dedicated software (MatInspector 2.2, Genomatix Software GmbH, Munich, Germany).

A series of truncated FGFR2 promoter fragments including −565 to +459, −143 to +459, −81 to +114, −81 to +58 and −81 to +5, were obtained by PCR and cloned into the pGL3-basic plasmid. The primer sequences used are reported in Table 2. All constructs were sequence verified.

Site-directed mutagenesis in the putative E2F1 binding region (+5 to +58) of the FGFR2 gene promoter was obtained respectively by *in vitro* synthesis of eight mutated sequences in which serial stretches of 7 nucleotides were replaced with a 5′-TTTTTTT-3′ stretch in different positions of the E2F1 responsive region (+5 to +11, +12 to +18, +19 to +25, +26 to +32, +33 to +39, +40 to +46, +47 to +53, +54 to +58) and by *in vitro* synthesis of seven mutated sequences, in each of which one nucleotide was replaced with a T in different positions of the E2F1 responsive element (+5 to +11). The mutated sequences were inserted into the pGL3-basic vector and then confirmed by DNA sequencing.

### Cell Transfection and Luciferase Reporter Assay

For the dual-luciferase reporter assay, HEK293 cells were seeded onto 24-well plates at a density of 2×10^5^ cells/well and co-transfected with 1 µg of the pGL3-basic based construct and 300 ng of the control pRL-TK plasmid for normalization of transfection efficiency. For the experiments evaluating promoter activation by E2F1, cells were also co-transfected with 100 ng of HA-E2F-1 wt-pRcCMV plasmid (Addgene plasmid 21667) [41] or with the same amount of empty pRcCMV vector (Invitrogen).

Transfections were carried out in serum free medium, using Lipofectamine 2000 reagent (Invitrogen) following manufacturer’s instructions. After 6 h, cells were treated with cytokines, where indicated. Luciferase activities were determined with Dual Luciferase Reporter Assay System (Promega) 24 h after treatment, according to manufacturer’s protocol. All transfections were conducted in triplicate.

### Chromatin Immunoprecipitation (ChIP)

Chromatin immunoprecipitation (ChIP) assays were performed using the EpiQuik Chromatin Immunoprecipitation Kit, following the protocol provided by Epigentek (Farmingdale, NY, USA). Briefly, HEK293 cells were harvested, cross-linked with 1% paraformaldehyde for 10 min at 25°C and quenched for 5 min with glycine. Cells were then washed with ice-cold PBS, resuspended in appropriate buffer containing a protease inhibitor mixture and sonicated three times for 10 s with a 1 min cooling period on ice. The extracted chromatin was immunoprecipitated with a rabbit polyclonal antibody to E2F1 (Santa Cruz). Positive (RNA polimerase II) and negative (rabbit IgG) control antibodies were used. Input and immunoprecipitated DNA were amplified by PCR using the following FGFR2 promoter-specific primers: 5′-GAAACGGCTCGGGTTTCAGTGG-3′ (forward), and 5′- CGAGTTGCGAAGGCTCAGAGC -3′ (reverse), which amplified the promoter region from -48 to +245.

### Statistical Analysis

Each set of experiments was repeated at least in triplicate, and standard deviation values were calculated. Student’s two-tailed *t*-test was used for statistical analysis, and *P*-values less than 0.05 were considered statistically significant.

## Results

### Effects of Proinflammatory Cytokines on Epithelial Cells Proliferation

**Figure 1:**
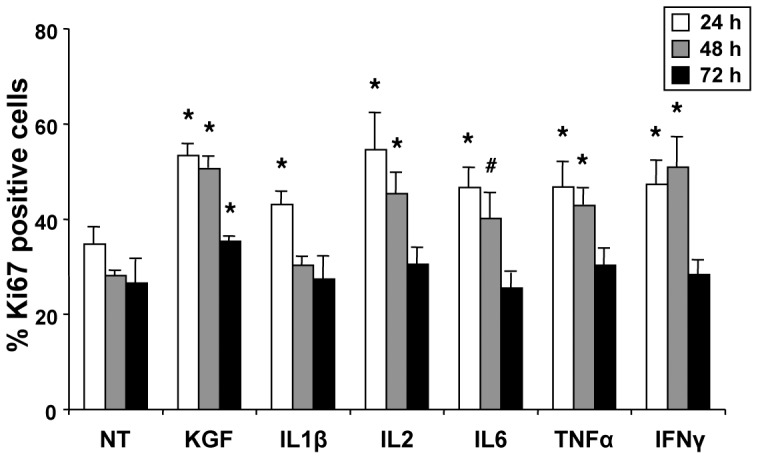
Effect of cytokines treatment on MCF-7 cell proliferation. Immunofluorescence analysis with a polyclonal antibody directed against Ki67 in MCF-7 cells that were left untreated, treated with 10 ng/ml KGF as a positive control or treated with 10 ng/ml IL1β, IL2, IL6, TNFα and IFNγ for 24, 48 and 72 h. The percentage of Ki67-positive cells was determined by counting the number of Ki67-positive nuclei *versus* total number of nuclei in ten different areas randomly taken from three different experiments. Error bars represent standard deviations. #*P*<0.05, *P<0.01.

A first series of experiments was aimed to investigate the effects of inflammatory cytokines on epithelial cells proliferation. MCF-7 cell line cultures were treated with IL1β, IL2, IL6, TNFα or IFNγ, and proliferative effects were assayed by evaluating staining with Ki67, a known marker for cell proliferation [42]–[43]. Cell cultures were treated with cytokines (10 ng/ml), assayed at 24, 48 or 72 h and compared to untreated cells. KGF at 10 ng/ml was used as positive control for cell proliferation. Figure 1 shows that an effect on MCF-7 cells was observed with all the treatments at 24 h, however it was more evident at 48 h for all cytokines but IL1β, then decreasing at 72 h, when only KGF retained a significant proliferative effect, as expected. In conclusion, all the tested cytokines seemed to stimulate MCF-7 proliferation, although to a different extent: IFNγ and IL2 appeared more effective, particularly at 48 h (1.81 and 1.61 fold, respectively, *P*<0.01), with an increase of the percentage of Ki67 positive cells comparable to that induced by KGF (1.80 fold, *P*<0.01), but also TNFα and IL6 induced MCF-7 proliferation (1.53 fold, *P*<0.01 and 1.43 fold, *P*<0.05, respectively). Conversely, IL1β was effective at 24 h (1.24 fold, *P*<0.01) but did not seem to induce a significant cell proliferation at 48 and 72 h, at least at this dose.

### Evaluation of the Effects of Cytokines Treatment on KGFR Expression in Epithelial Cells

Since activation of the KGF/KGFR axis is known to represent a major pathway to induce epithelial cells proliferation, and an upregulation of KGFR expression has been reported in inflammatory lesions [27]–[28], we assayed whether treatment with the various cytokines was able to affect the expression of KGFR. MCF-7 cells were treated for a maximum of 72 h and collected at intervals to analyze KGFR expression both at mRNA level, by means of quantitative real-time PCR, and at protein level, through Western blot.

As concerning KGFR protein expression, it is noteworthy that anti-Bek antibody used in these experiments could not distinguish the expression of KGFR and FGFR2-IIIc, since it recognizes intracellular domain that are commonly involved in both isoforms. Nevertheless, it has been previously demonstrated that MCF-7 cells express both FGFR2 isoforms, but the amount of IIIb is greatly higher than IIIc [44]–[45]. Therefore, the contribution of FGFR2-IIIc can be considered negligible in the experiments with MCF-7 cells.

**Figure 2:**
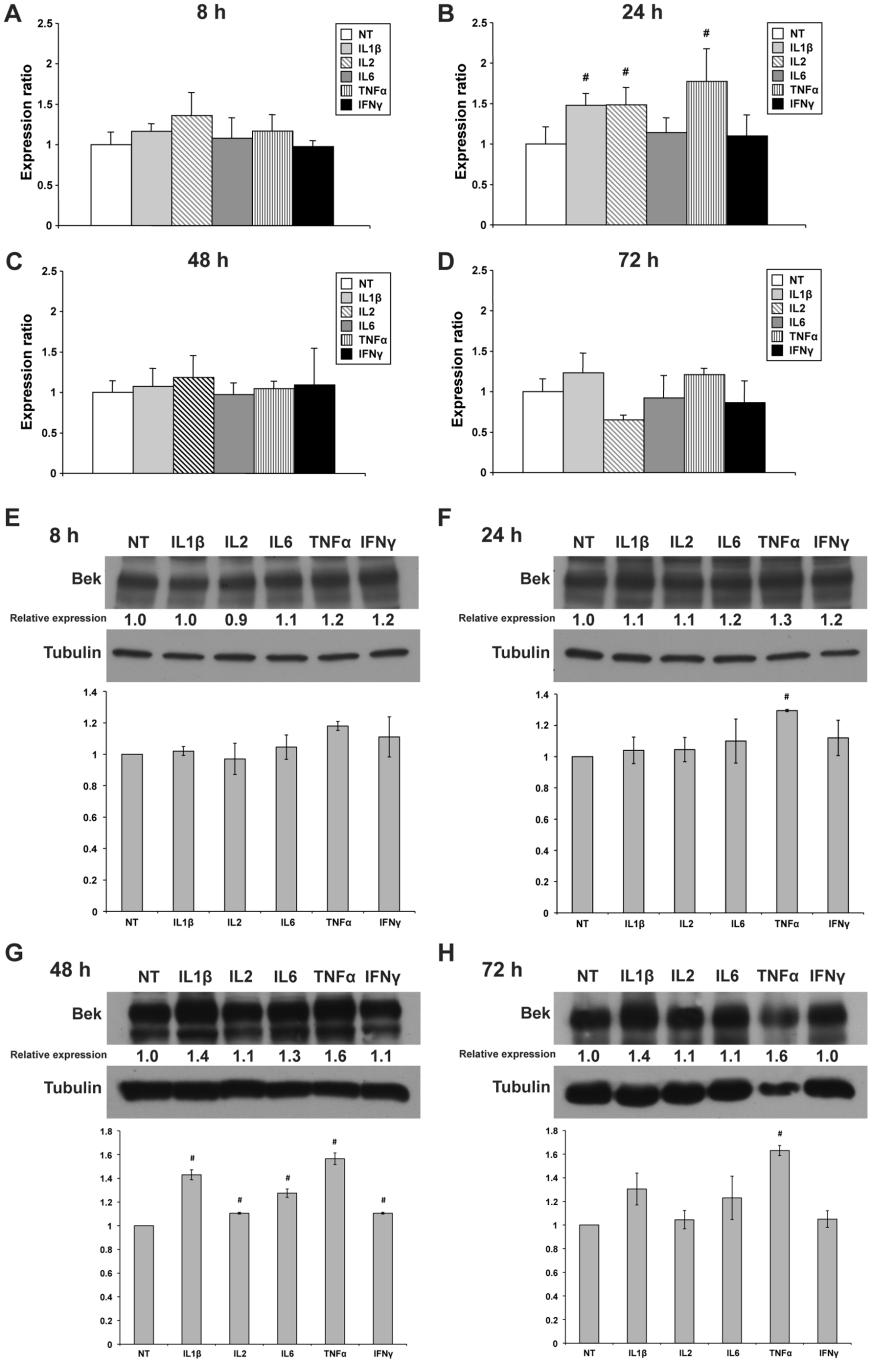
Effect of cytokines on the regulation of KGFR mRNA and protein expression in MCF-7 cells. (**A–D**) Quantitative real-time PCR analysis of KGFR mRNA expression in MCF-7 cells following treatment with 10 ng/ml IL1β, IL2, IL6, TNFα or IFNγ for 8, 24, 48 and 72 h. Relative KGFR mRNA levels are shown as fold value of the level of KGFR mRNA in untreated cells. Each experiment was performed in triplicate, and mRNA levels were normalized to GAPDH mRNA expression. Error bars represent standard deviations. #*P*<0.05. (**E–H**) Western blot analysis of KGFR protein levels in MCF-7 cells untreated or treated with 10 ng/ml IL1β, IL2, IL6, TNFα or IFNγ for 8, 24, 48 and 72 h. KGFR protein expression was evaluated by blotting with an anti-Bek antibody. Western blot with anti-Tubulin antibody was used as loading control. The images are representative of at least three independent experiments. The intensity of the bands was evaluated by densitometric analysis, normalized and reported as relative expression with respect to the untreated cells. Densitometric analysis was also performed for each experiment and reported as a graph. Error bars represent standard deviations. #*P*<0.05.

As observed in Figure 2, no significant increase in KGFR mRNA (Figure 2A) and protein (Figure 2E) expression was evident at 8 h of treatment with IL1β, IL2, IL6, TNFα or IFNγ. At 24 h, TNFα and, to a lesser extent, IL1β as well as IL2 induced a significant increase of KGFR expression at transcriptional level (1.77, 1.48 and 1.48 fold, respectively, *P*<0.05) (Figure 2B) that was still not striking at protein level (Figure 2F). However, the transcriptional activation reflected in a detectable increase in KGFR protein level expression in IL1β- and TNFα-treated cell cultures after 48 (1.4 and 1.6 fold, respectively) and 72 h (1.4 and 1.6 fold, respectively) (Figure 2G, H).

**Figure 3:**
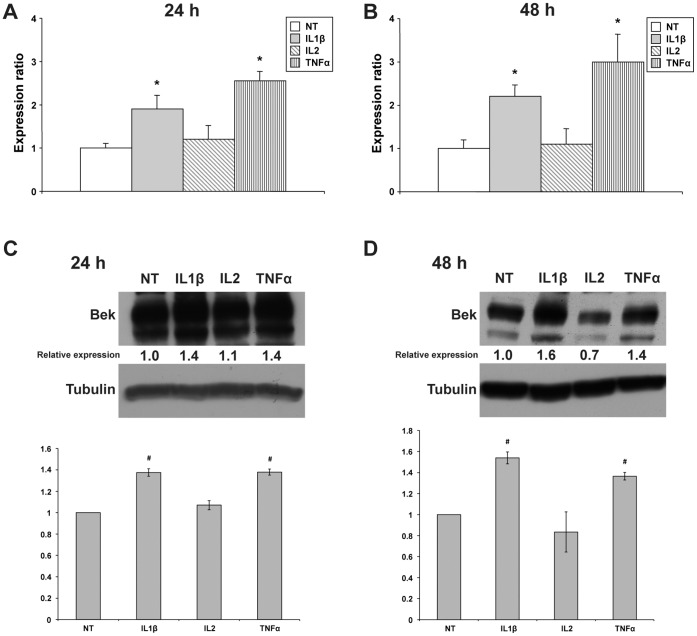
Effect of higher doses of cytokines on KGFR mRNA and protein expression in MCF-7 cells. (**A–B**) Quantitative real-time PCR analysis of KGFR mRNA expression in MCF-7 cells following treatment with 100 ng/ml IL1β, IL2 or TNFα for 24 and 48 h. Relative KGFR mRNA levels are shown as fold value of the level of KGFR mRNA in untreated cells. Each experiment was performed in triplicate, and mRNA levels were normalized to GAPDH mRNA expression. Error bars represent standard deviations. *P<0.01. (**C–D**) Western blot analysis of KGFR protein levels in MCF-7 cells untreated or treated with 100 ng/ml IL1β, IL2 and TNFα for 24 and 48 h. KGFR protein expression was evaluated by blotting with an anti-Bek antibody. Western blot with anti-Tubulin antibody was used as loading control. The images are representative of at least three independent experiments. The intensity of the bands was evaluated by densitometric analysis, normalized and reported as relative expression with respect to the untreated cells. Densitometric analysis was also performed for each experiment and reported as a graph. Error bars represent standard deviations. #*P*<0.05.

To highlight the effects on KGFR expression, we focused our attention on TNFα, IL1β and IL2, using higher concentrations of these cytokines (100 ng/ml). As concerning TNFα treatment, we performed experiments with both 50 and 100 ng/ml and we found comparable results in terms of FGFR2 expression (Figure S1), with a slightly increased toxicity at 100 ng/ml. For this reason, we adopted the highest dose (100 ng/ml) for Western blot and real-time PCR experiments, to maintain the same doses used for the other cytokines. In these conditions, at 24 and 48 h both IL1β and TNFα induced an increase in KGFR mRNA expression, with a maximum of 2.2 and 3.0 fold, respectively (Figure 3A, B; *P*<0.01). In this set of experiments, KGFR protein levels reflected the course of mRNA levels, with a significant increase following IL1β and TNFα treatment at 24 (1.4 and 1.4 fold, respectively) and 48 h (1.6 and 1.4 fold, respectively) (Figure 3C, D). It has to be remarked that IL2 under these conditions did not confirm its efficacy in inducing KGFR upregulation, probably due to its toxicity at this concentration.

Taken altogether, these experiments seem to indicate that one of the effects induced by cytokines released in the inflammatory environment, and in particular by IL1β and TNFα, is the upregulation of KGFR expression, a key element in wound healing.

### Role of Cytokines in the Modulation of KGF and FGFR2-IIIc Expression in Mesenchymal Cells

**Figure 4:**
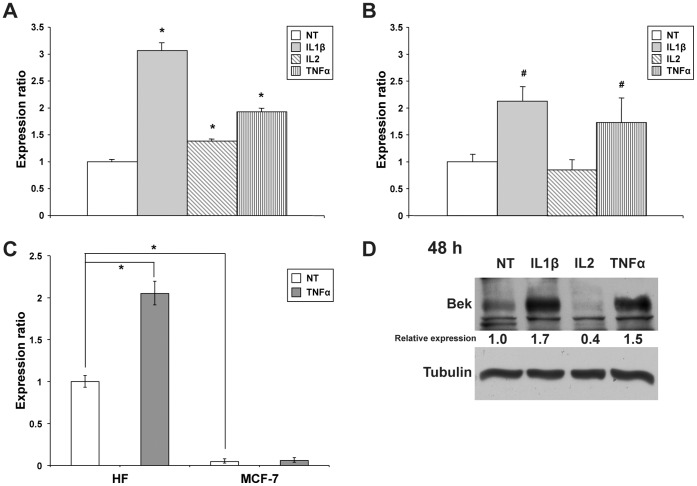
Effect of cytokines on KGF and FGFR2-IIIc mRNA expression in human primary fibroblasts. (**A**) Quantitative real-time PCR analysis of KGF mRNA expression following treatment with 100 ng/ml IL1β, IL2 or TNFα for 24 h. Relative KGF mRNA levels are shown as fold value of the level of KGF mRNA in untreated cells. (**B**) Quantitative real-time PCR analysis of FGFR2-IIIc mRNA expression following treatment with 100 ng/ml IL1β, IL2 or TNFα for 24 h. Relative FGFR2-IIIc mRNA levels are shown as fold value of the level of FGFR2-IIIc mRNA in untreated cells. Each experiment was performed in triplicate, and mRNA levels were normalized to GAPDH mRNA expression. Error bars represent standard deviations. #*P*<0.05, *P<0.01. (**C**) Quantitative real-time PCR analysis to compare the amount of FGFR2-IIIc mRNA expression in HF and in MCF-7 cells, both untreated or treated with 100 ng/ml TNFα for 24 h. Relative FGFR2-IIIc mRNA levels are shown as fold value of the level of FGFR2-IIIc mRNA in untreated HF cells. Each experiment was performed in triplicate, and mRNA levels were normalized to GAPDH mRNA expression. Error bars represent standard deviations. *P<0.01. (**D**) Western blot analysis of FGFR2-IIIc protein levels in HF cells untreated or treated with 100 ng/ml IL1β, IL2 and TNFα for 48 h. FGFR2-IIIc protein expression was evaluated by blotting with an anti-Bek antibody. Western blot with anti-Tubulin antibody was used as loading control. The intensity of the bands was evaluated by densitometric analysis, normalized and reported as relative expression with respect to the untreated cells.

The paracrine interactions between keratinocytes and fibroblasts that underlie the healing process are strictly regulated by KGF/KGFR axis. Therefore, to confirm the effects of the above-mentioned cytokines on the expression of KGF mRNA levels, we set up primary cultures of human fibroblasts. As shown in Figure 4A, all the tested cytokines were able to stimulate KGF mRNA expression. In particular, IL1β increased the RNA level up to 3.07 fold (*P*<0.01), although also TNFα (1.93 fold, *P*<0.01) and, to a lesser extent, IL2 (1.39 fold, *P*<0.01) upregulated KGF expression. The dual effect of IL1β and TNFα in stimulating the expression of both ligand and its receptor (KGF and KGFR) highlights the role of these cytokines in the physiological processes that involve the KGF/KGFR signaling, with particular regard to epithelial cell proliferation and wound healing.

Since the FGFR2 gene gives rise to KGFR in epithelial cells and to its alternative splicing transcript variant FGFR2-IIIc in mesenchymal cells, we assessed the possible modulation of FGFR2-IIIc expression by cytokines treatment in primary cultures of human fibroblasts. The expression of FGFR2-IIIc mRNA in cells treated with IL1β, IL2 or TNFα was measured by quantitative real-time PCR, and compared to that of untreated cells. As shown in Figure 4B, both IL1β and TNFα were able to induce a significant increase in FGFR2-IIIc expression (2.12 and 1.70 fold, respectively, *P*<0.05), thus confirming an effect on FGFR2 gene transcription in both tissues.

Moreover, we performed quantitative real-time PCR experiments to compare the amount of FGFR2-IIIc mRNA in MCF-7 and human fibroblasts (HF), both untreated or treated with TNFα. The data obtained (Figure 4C) were in accordance with previous literature [44]–[45] as concerning the very low levels of FGFR2-IIIc expression in untreated MCF-7 cells (0.05 fold, *P*<0.01 *versus* untreated HF cells). Therefore, also the effect of TNFα treatment on FGFR2-IIIc in MCF-7 cells is negligible, while FGFR2-IIIc expression is affected by TNFα in HF cells, as previously demonstrated (2.1 fold, *P*<0.01 *versus* untreated HF cells).

To assess the effect of these cytokines on FGFR2-IIIc also at protein level, we performed Western blot analysis on primary cultured fibroblasts treated or not with IL1β, IL2 and TNFα. The results obtained (Figure 4D) pointed out that FGFR2-IIIc protein levels reflected those of its mRNA, with an increase after IL1β and TNFα treatment at 48 h (1.7 and 1.5 fold increase, respectively). Similarly to what observed in real-time PCR and to what previously reported for FGFR2-IIIb in MCF-7 cells (Figure 3), IL2 treatment was not able to induce FGFR2-IIIc upregulation.

### Role of Cytokines in FGFR2 Promoter Activation

**Figure 5:**
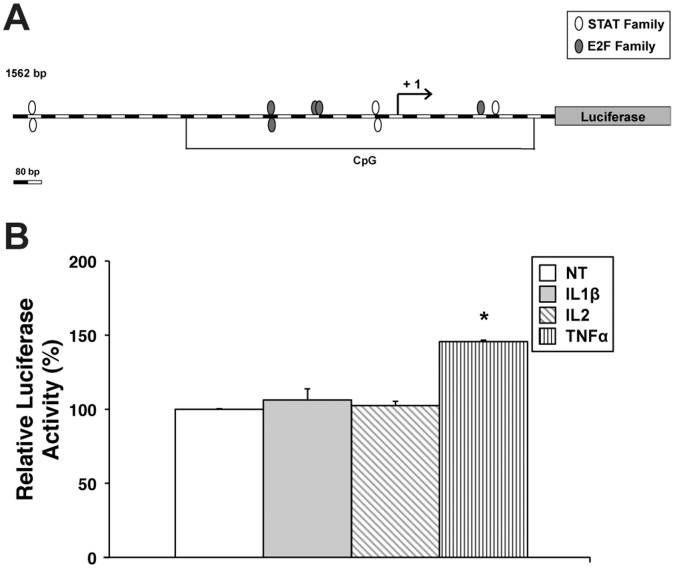
Role of TNFα in the stimulation of FGFR2 promoter activity. (**A**) Schematic representation of the FGFR2 promoter construct, in which a 1.5 kb cassette of FGFR2 gene around the transcription initiation site is linked to the luciferase reporter gene. Putative binding sites for STAT and E2F transcription factors families in the promoter sequence are shown as white or grey ovals, respectively. (**B**) Luciferase reporter assays were performed in HEK293 cells. The recombinant pGL3-basic-1.5 kb FGFR2 promoter (−1139/+459) construct was transfected into HEK293 cells. 6 h after transfection, cells were left untreated or treated with 100 ng/ml IL1β, 100 ng/ml IL2 or 50 ng/ml TNFα, and luciferase activities were determined 24 h after treatment. Luciferase reporter assay data are expressed as percentage of control (untreated cells) and represent the means of three separate experiments after correcting for differences in transfection efficiency by pRL-TK activities. Error bars represent standard deviations. *P<0.01.

The observation of the consistent upregulation of human KGFR and FGFR2-IIIc mRNA expression following treatment with TNFα and IL1β led us to investigate the molecular mechanisms involved in the regulation of FGFR2 gene transcription. Thus far, little is known about the promoter region of the human FGFR2 gene as well as the mechanisms involved in the control of its expression. Our first approach was the analysis of the structure of the hypothetic promoter region searching for known consensus motifs. Our attention was focused on the STAT family members, which are known to be involved in the signal transduction of most of the cytokines [46], as well as on known E2F responding motifs, since E2F family members have been previously reported to be involved in the regulation of other FGFR genes [37], [39]. In Figure 5A it is reported the position of putative sites located in a region spanning 1.5 kb around the transcriptional initiation site. To verify whether the analyzed region contains elements that are stimulated by cytokines, we cloned this fragment in a luciferase reporter vector and set up a transactivation assay. The plasmid harboring the entire 1.5 kb region was transfected in HEK293 cells, an epithelial cell line which has been shown to represent a good cellular model for transfection experiments. After transfection, cell cultures were treated with the three cytokines that proved to be more effective in our previous experiments. As shown in Figure 5B, no significant increase in luciferase activity was observed following treatment with IL1β and IL2, compared to untreated cells. However, TNFα seemed to be effective in inducing promoter activation, leading up to a 145% luciferase activity with respect to untreated cells (*P*<0.01).

Mechanisms involved in TNFα-mediated activation of gene transcription have been previously studied [47] and it has been shown that TNFα treatment induced the activation of E2F family. Furthermore, a potential role of E2F1 in the activation of FGFR transcription has been previously reported for the murine homolog of the FGFR2 gene [37], as well as for the human FGFR1 gene [39]. Thus, we set up experiments to analyze whether activation of the human FGFR2 gene may involve the E2F1 transcription factor.

**Figure 6:**
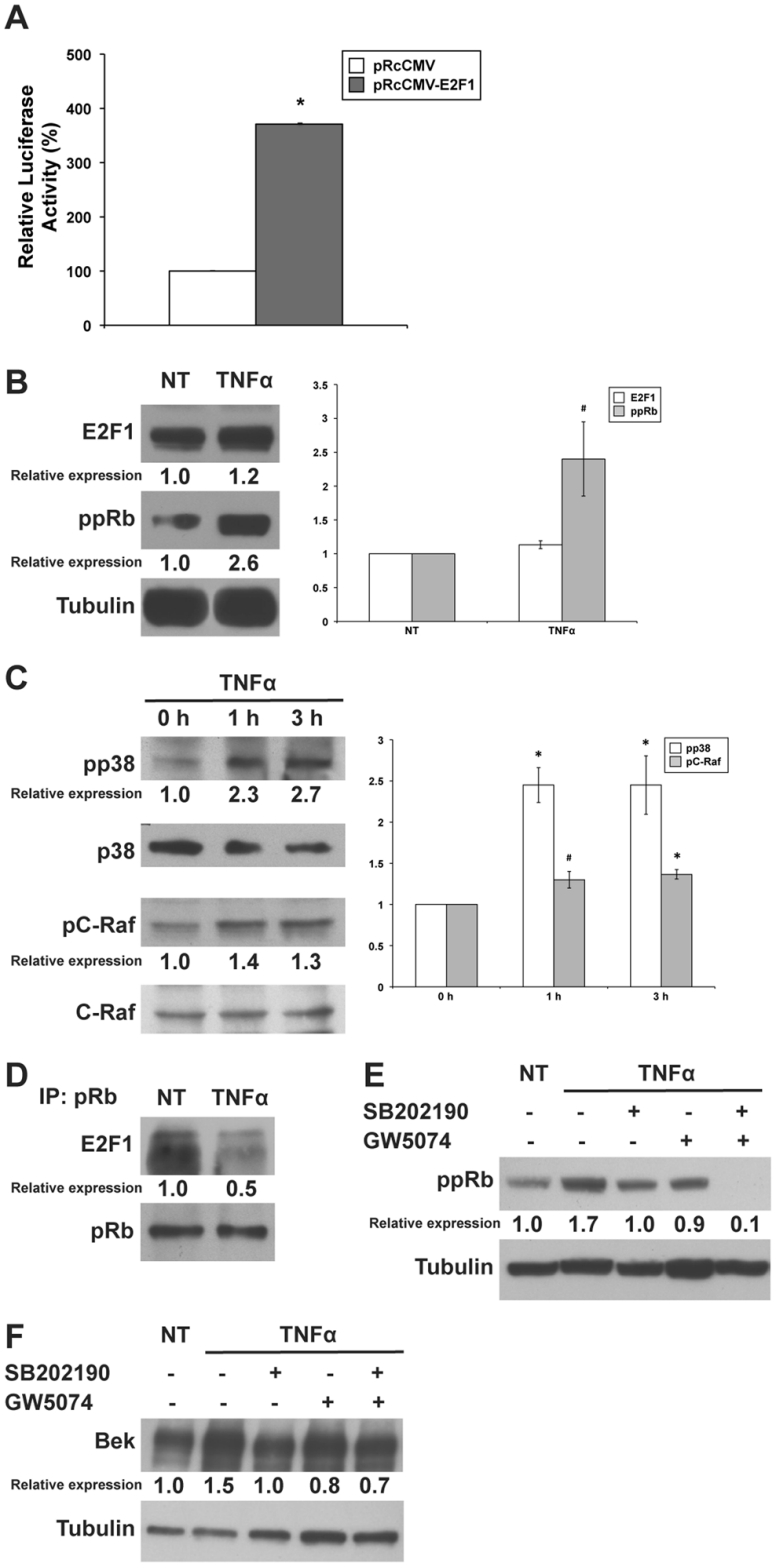
Role of E2F1 in TNFα-induced stimulation of FGFR2 promoter. (**A**) Luciferase reporter assays were performed in HEK293 cells. The recombinant pGL3-basic-1.5 kb FGFR2 promoter (−1139/+459) construct was co-transfected into HEK293 cells with pRcCMV (empty vector) or pRc-CMV-E2F1, and luciferase activities were determined 24 h after trasfection. Data are expressed as percentage of control (cells transfected with pRcCMV alone) and represent the means of three separate experiments after correcting for differences in transfection efficiency by pRL-TK activities. Error bars represent standard deviations. *P<0.01. (**B**) Western blot analysis of E2F1 protein levels and pRb phosphorylation status in MCF-7 cells untreated or treated with 100 ng/ml TNFα for 3 h. E2F1 protein expression was assessed by blotting with an anti-E2F1 polyclonal antibody. pRb phosphorylation was evaluated by blotting with an anti-phospho-pRb antibody. Tubulin was used as loading control. The images are representative of at least three independent experiments. The intensity of the bands was evaluated by densitometric analysis, normalized and reported as relative expression with respect to the untreated cells. Densitometric analysis was also performed for each experiment and reported as a graph. Error bars represent standard deviations. #*P*<0.05. (**C**) Western blot analysis of p38 and C-Raf phosphorylation status in MCF-7 cells untreated or treated with 100 ng/ml TNFα for 1 and 3 h. p38 and C-Raf phosphorylation was evaluated by blotting with anti-phospho-p38 and anti-phospho-C-Raf antibodies, respectively. Western blot with anti-p38 or anti-C-Raf antibodies, respectively, was used as loading control. The images are representative of at least three independent experiments. The intensity of the bands was evaluated by densitometric analysis, normalized and reported as relative expression with respect to the untreated cells. Densitometric analysis was performed for each experiment and reported as a graph. Error bars represent standard deviations. #*P*<0.05, *P<0.01. (**D**) Co-immunoprecipitation assay was performed to study *in vivo* interaction between pRb and E2F1 proteins. MCF-7 cells, untreated or treated with 100 ng/ml TNFα, were immunoprecipitated with anti-pRb antibody and blotted with anti-E2F1 antibody. Western blot with anti-pRb antibody was used as loading control. (**E**) Western blot analysis of pRb phosphorylation status in MCF-7 cells untreated or treated with 100 ng/ml TNFα for 3 h, alone or in the presence of the p38 inhibitor SB202190 (10 µM), the C-Raf inhibitor GW5074 (1 µM ) or both of them. pRb phosphorylation was evaluated by blotting with an anti-phospho-pRb antibody. Tubulin was used as loading control. The intensity of the bands was evaluated by densitometric analysis, normalized and reported as relative expression with respect to the untreated cells. (**F**) Western blot analysis of KGFR protein levels in MCF-7 cells untreated or treated with 100 ng/ml TNFα for 48 h, alone or in the presence of the p38 inhibitor SB202190 (10 µM), the C-Raf inhibitor GW5074 (1 µM) or both of them. KGFR protein expression was evaluated by blotting with an anti-Bek antibody. Western blot with anti-Tubulin antibody was used as loading control. The intensity of the bands was evaluated by densitometric analysis, normalized and reported as relative expression with respect to the untreated cells.

We transfected our reporter plasmid in HEK293 cells together with a plasmid that drives the expression of the E2F1 protein (pRcCMV-E2F1). As shown in Figure 6A, E2F1 induced a strong activation of the FGFR2 promoter, reaching 371% of activation when compared to cells co-transfected with the empty vector (pRcCMV) (*P*<0.01).

It is known that the hypo-phosphorylated form of pRb binds to E2F1, thus preventing its nuclear translocation and activation of target genes [48], while hyper-phosphorylation of pRb stimulates the release of transcriptionally active E2F1. Therefore, we decided to verify the phosphorylation status of pRb in HEK293 cells following treatment with TNFα. As observed in Figure 6B, TNFα strongly induced the phosphorylation of pRb (2.6 fold). At the same time, Western blot analysis with an anti-E2F1 antibody showed that total protein levels of E2F1 were only slightly affected by TNFα treatment (1.2 fold, Figure 6B).

The regulation of E2F activity by pRb is dependent on a number of other factors that regulate the function of Rb family members. For instance, cdk-cyclin complex has the ability to hyperphosphorylate the Rb family members [49]. Moreover, it has been previously demonstrated that increase in Rb phosphorylation can be mediated via the p38 mitogen-activated protein kinase [50]–[52].

For this reason, since previous works showed that TNFα is able to activate p38 kinase [53]–[54], we assessed the effect of TNFα on p38 phosphorylation in MCF-7 cells. As demonstrated in Figure 6C, TNFα was able to induced the phosphorylation of p38 at both 1 and 3 h (2.3 and 2.7 fold, respectively).

It is known that also C-Raf (Raf-1) kinase binds to Rb and phosphorylates Rb in the early G1 phase, thus allowing the subsequent hyper-phosphorylation and inactivation of Rb, with the release of E2F1 [55].

Since TNFα has been shown to activate Raf/MEK/ERK pathway and facilitate Rb/C-Raf interaction, we performed a Western blot analysis with an anti-phospho C-Raf antibody, showing that TNFα treatment at both 1 and 3 h was able to induce a slight increase of C-Raf phosphorylation (1.4 and 1.3 fold, respectively, Figure 6C).

To further demonstrate the dissociation between pRb and E2F1, we performed a co-immunoprecipitation experiment. As shown in Figure 6D, in untreated cells we were able to co-immunoprecipitate pRb and E2F1, while in TNFα-treated cells immunoprecipitated with pRb, E2F1 amount was greatly reduced (0.5 fold), thus confirming its release from pRb. Therefore, it is conceivable to hypothesize that TNFα-induced transcription of the FGFR2 human gene is mediated through the release of E2F1.

To further clarify the role of p38 and C-Raf phosphorylation in pRb/E2F1 pathway activation and in FGFR2 expression, we assessed the effect of TNFα on pRb phosphorylation in MCF-7 cells pretreated or not with SB202190, a p38 inhibitor, with GW5074, a C-Raf inhibitor, or with a combination of them. As shown in Figure 6E, TNFα-induced phosphorylation of pRb (1.7 fold) was reduced to the levels of untreated cells by treatment with both SB202190 and GW5074 (1.0 and 0.9 fold, respectively). Moreover, the combined treatment with both inhibitors completely abolished pRb phosphorylation (0.1 fold), thus confirming the contribution of both pathways to Rb/E2F activation. Then, we also assessed the expression of KGFR in MCF-7 cells treated with TNFα, in the presence of the p38 inhibitor SB202190, the C-Raf inhibitor GW5074, or both. The results obtained, showed in Figure 6F, indicated that inhibition of p38 or C-Raf signaling was able to prevent TNFα-dependent KGFR upregulation (1.0 and 0.8 fold, respectively), as well as inhibition of both pathways (0.7 fold).

**Figure 7:**
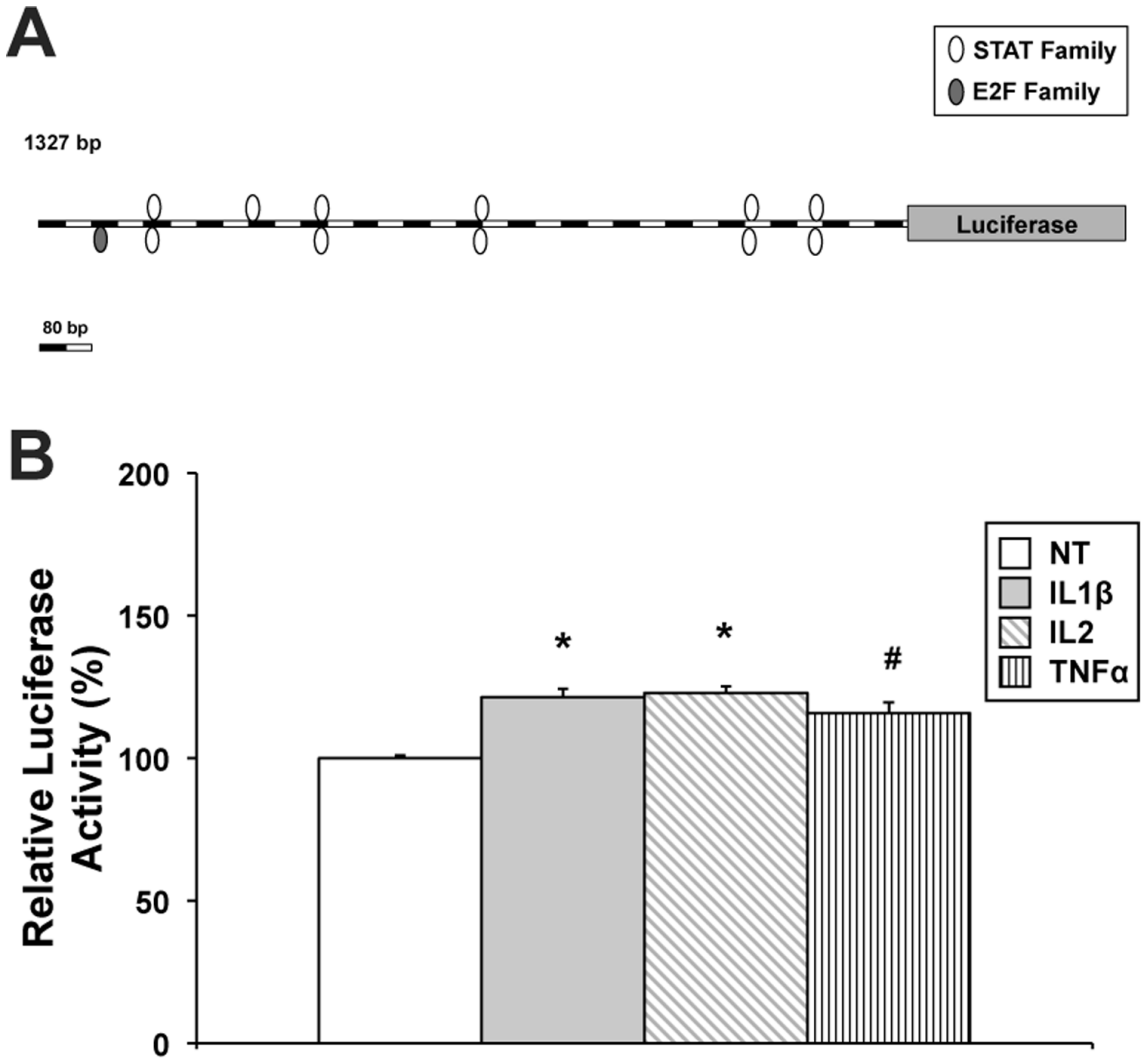
Role of proinflammatory cytokines in the stimulation of FGFR2 promoter activity. (**A**) Schematic representation of the second FGFR2 promoter construct, in which a 1.3 kb cassette of FGFR2 gene upstream of the previously used 1.5 kb cassette is linked to the luciferase reporter gene. Putative binding sites for STAT and E2F transcription factors families in the promoter sequence are shown as white or grey ovals, respectively. (**B**) Luciferase reporter assays were performed in HEK293 cells. The recombinant pGL3-basic-1.3 kb FGFR2 promoter (−2235/−909) construct was transfected into HEK293 cells. 6 h after transfection, cells were left untreated or treated with 100 ng/ml IL1β, 100 ng/ml IL2 or 50 ng/ml TNFα, and luciferase activities were determined 24 h after treatment. Luciferase reporter assay data are expressed as percentage of control (untreated cells) and represent the means of three separate experiments after correcting for differences in transfection efficiency by pRL-TK activities. Error bars represent standard deviations. #*P*<0.05, *P<0.01.

To verify if the three cytokines, especially IL1β and IL2, should be able to act on different sites of the FGFR2 promoter, we cloned in a luciferase reporter vector a promoter region of 1.3 kb spanning upstream the 1.5 kb region, which contains much more putative sites belonging to the STAT family (Figure 7A). The plasmid harboring this 1.3 kb region was transfected in HEK293 cells, and cell cultures were treated with IL1β, IL2, and TNFα. As documented in Figure 7B, all the three cytokines were able to increase luciferase activity if compared to untreated cells. In particular, IL1β determined 121% activation (P<0.01), and IL2 determined 123% activation (P<0.01). TNFα resulted to be less effective in inducing the activation of this promoter construct (116% luciferase activity with respect to untreated cells, *P*<0.05), probably due to the presence of only one E2F putative site.

### Identification of E2F1 Responsive Sequences in the Human FGFR2 Promoter

As previously mentioned, we searched for putative E2F binding sequences in the 1.5 kb human FGFR2 promoter region, from −1103 to +459, by means of a dedicated software (MatInspector 2.2), finding out 5 possible binding sites, as reported in Figure 5A (grey ovals).

**Figure 8:**
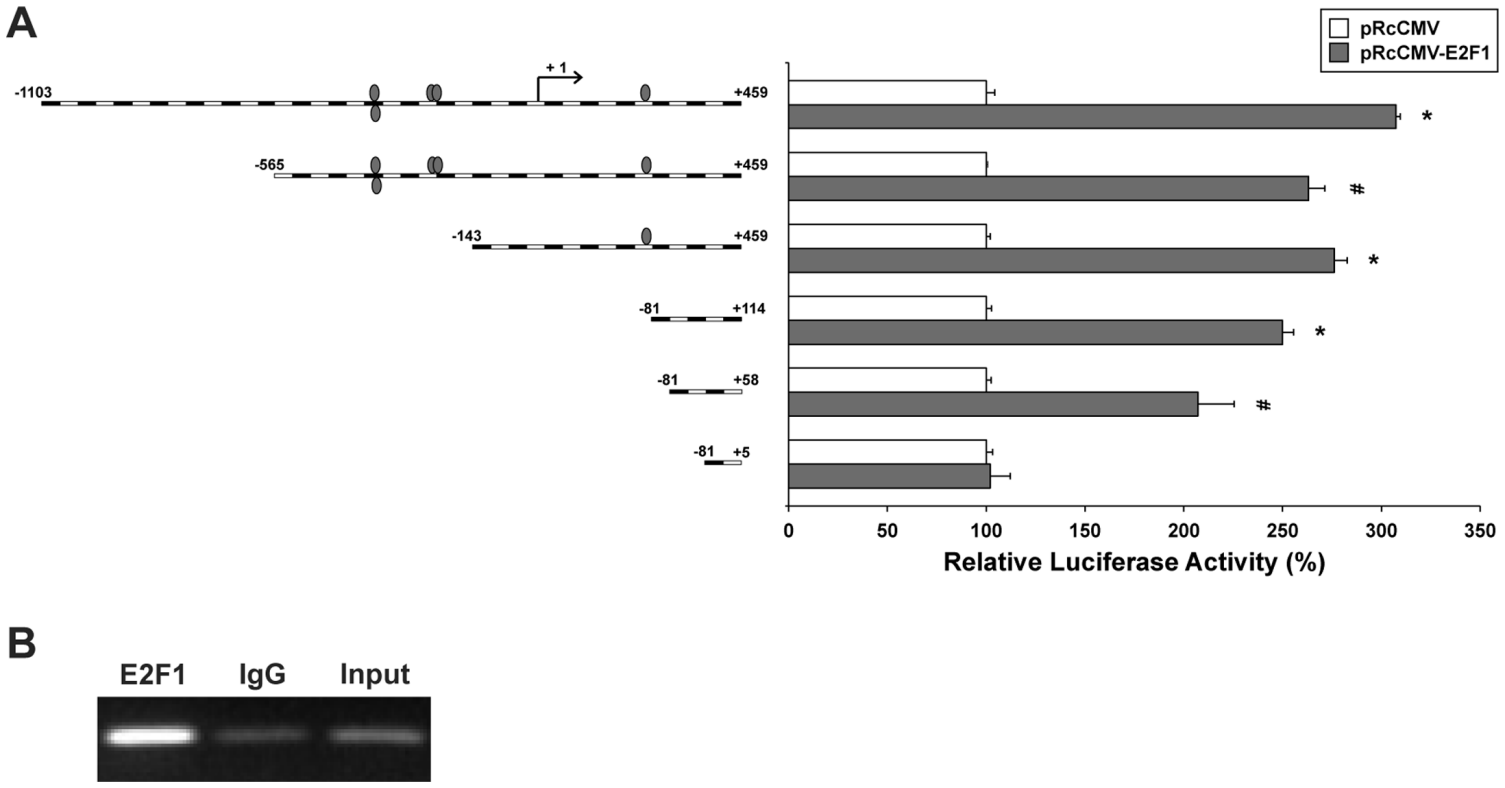
Identification of E2F1-responsive region in the FGFR2 promoter. (**A**) Truncation analysis of FGFR2 promoter activity in HEK293 cells. Cells were co-transfected with different pGL3-basic-FGFR2 promoter truncated constructs and pRcCMV (empty vector) or pRc-CMV-E2F1, and luciferase activities were determined 24 h after transfection. Constructs with different FGFR2 promoter lengths are depicted along the left. Putative binding sites for E2F transcription factors family in the promoter sequence are shown as grey ovals. Luciferase reporter assay data are expressed as percentage of control (cells transfected with pRcCMV alone) and represent the means of three separate experiments after correcting for differences in transfection efficiency by pRL-TK activities. Error bars represent standard deviations. #*P*<0.05, *P<0.01. (**B**) Lysates of HEK293 cells were subjected to ChIP with indicated antibodies (E2F1 or IgG). Immunoprecipitated DNA and input DNA were subjected to PCR amplification of the FGFR2 promoter region between −48 and +245. The image is representative of three independent experiments.

To verify the functionality of these putative E2F binding sequences, we generated a series of truncated FGFR2 promoter fragments linked to a luciferase reporter gene. Each of these constructs was co-transfected with pRcCMV-E2F1 or pRcCMV empty vector, respectively. Figure 8A shows that the first deleted construct (−565/+459), in which all the putative E2F sites were conserved, maintained a consistent responsiveness to E2F1 (263%, *P*<0.05), with a disregarding reduction compared to the full-length promoter (307%, *P*<0.01). Moreover, a similar activation (276%, *P*<0.01) was observed with a shorter construct (−143/+459) that retains only one of the five putative E2F binding sites.

Also the −81/+114 construct, lacking the last potential E2F binding site, retained a significant responsiveness (250%, *P*<0.01). A previous study carried out on the mouse FGFR2 gene [37] identified a non-canonical E2F1 responsive motif that has a positional homology with a stretch located between +70/+77 in human FGFR2, although the sequence is not conserved. Therefore, we generated a further truncated construct (−81/+58), which resulted to be still activated by E2F1, even though at a slightly reduced level (207%, *P*<0.05). Finally, we found that a shorter fragment (−81/+5) lost E2F1 responsiveness, suggesting that the E2F binding region lies within the +5/+58 sequence.

To substantiate the evidence that E2F1 is physically recruited to the endogenous human FGFR2 promoter, we performed chromatin immunoprecipitation (ChIP) using an anti-E2F1 antibody. Using DNA fragments precipitated with anti-E2F1 as templates, a pair of primers was designed to amplify the promoter region from −48 to +245, which encompasses the E2F-responsive fragment identified in the previous luciferase assays. As shown in Figure 8B, the amplification of the selected region was consistently increased when the DNA fragments were immunoprecipitated with anti-E2F1, compared to the relevant input DNA, thus confirming that this promoter region binds to the E2F1 protein.

### Characterization of the Minimal E2F1 Responsive Element

**Figure 9:**
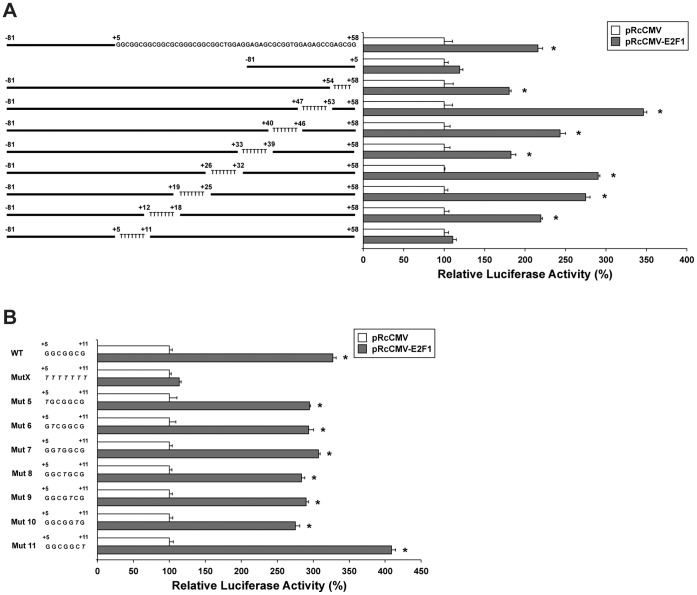
Mutation analysis of FGFR2 promoter activity in HEK293 cells. (**A**) Constructs in which pGL3-basic was linked to different FGFR2 promoter regions (−81/+58 or −81/+5) or to synthetic sequences with sequentially mutated 7 nucleotides stretches in the E2F1 responsive region (+5/+58) were co-transfected with pRcCMV empty vector or with pRcCMV-E2F1 into HEK293 cells, and luciferase activities were determined 24 h after transfection. Normal and mutated constructs of FGFR2 promoter are depicted along the left. Luciferase reporter assay data are expressed as percentage of control (cells transfected with pRcCMV alone) and represent the means of three separate experiments after correcting for differences in transfection efficiency by pRL-TK activities. Error bars represent standard deviations. *P<0.01. (**B**) Constructs in which pGL3-basic was linked to FGFR2 promoter regions containing the wild-type +5/+11 sequence (WT), the mutated +5/+11 stretch (MutX) or seven mutated sequences, in each of which one nucleotide was replaced with a T in different positions of the +5/+11 sequence, were co-transfected with pRcCMV empty vector or with pRcCMV-E2F1 into HEK293 cells, and luciferase activities were determined 24 h after transfection. Normal and mutated constructs of FGFR2 promoter are depicted along the left. Luciferase reporter assay data are expressed as percentage of control (cells transfected with pRcCMV alone) and represent the means of three separate experiments after correcting for differences in transfection efficiency by pRL-TK activities. Error bars represent standard deviations. *P<0.01.

Since genetic analysis showed that no consensus E2F1 binding motifs are present in the +5/+58 region, we hypothesized that transcription of the human FGFR2 gene might be regulated by E2F1 via a non-canonical E2F binding sequence, in keeping with similar observations reported on mouse homolog [37]. Thus, we set up experiments of site-directed mutagenesis, by synthesizing eight different −81/+58 promoter fragments bearing mutations of serial stretches of 7 nucleotides in the region from +5 to +58 (Figure 9A). Mutants were analyzed by luciferase reporter assay in HEK293 cells, following co-transfection with pRcCMV-E2F1 or pRcCMV empty vector. The first mutated construct, +54 to +58, showed an activation of 180%, lower than the wild-type construct (216%) but still statistically significant (*P*<0.01). The +47/+53 and +40/+46 mutated constructs were consistently activated by E2F1 (346% and 243%, respectively, *P*<0.01), while the +33/+39 plasmid was stimulated to an activation of 183% (*P*<0.01). The +26/+32, +19/+25 and +12/+18 mutated constructs retained a significant activation of luciferase reporter gene (290%, 275% and 219%, respectively, *P*<0.01), but when the +5/+11 sequence was mutated, E2F1 was not able to transactivate the reporter, leading us to postulate that this stretch of 7 nucleotides (5′-GGCGGCG-3′) may represent the non-canonical E2F1 binding motif within the human FGFR2 gene.

So, we performed a further site-directed mutagenesis on the +5/+11 sequence, by *in vitro* synthesis of seven mutated sequences, in each of which one nucleotide was replaced with a T in different positions (Figure 9B). Mutants were analyzed by luciferase reporter assay in HEK293 cells, following co-transfection with pRcCMV-E2F1 or pRcCMV empty vector. The activity of the construct bearing the WT +5/+11 sequence and of the construct with all the sequence mutated (327% and 114%, respectively) were unchanged with respect to the previous experiments (see Figure 9A). All the single-nucleotide-mutated constructs were still consistently responsive to E2F1 (ranging from 275% to 405%, *P*<0.01). Such results suggest that none of the single nucleotides of the +5/+11 sequence is essential for E2F1 binding to the FGFR2 promoter.

## Discussion

The mechanisms underlying the complex network between epithelial and mesenchymal cells are not fully understood, but FGFs and their cognate receptors family FGFRs are recognized as key elements [17], [56]–[59].

Here we analyzed the effects of inflammatory cytokines in inducing the expression of both FGFR2 and KGF genes in epithelial or mesenchymal cells. The capacity of cytokines to stimulate both KGF and KGFR expression, thus activating a paracrine loop, is intriguing especially in relation with physiological and pathological processes that involve dermal-epidermal interactions. While activation of KGF synthesis by cytokines has been previously reported [18]–[21], and it is confirmed in our study, very few information are known about the regulation of human FGFR2 gene. To our knowledge, the present study is the first evidence of the role of proinflammatory cytokines in the upregulation of FGFR2 mRNA expression and gene transcription. Here we observed that FGFR2 gene expression can be induced in both epithelial cell lines and human primary fibroblasts treated with IL1β or TNFα, giving rise to KGFR and FGFR2-IIIc respectively, as a result of the histotypic alternative splicing. This observation seems to indicate that the local recruitment of inflammatory mediators during wound healing promotes the activation of KGF/KGFR signaling, thus contributing to epithelial repair.

Few studies have been previously carried out on the molecular mechanisms that regulate human FGFR2 expression. Quite recently, it has been reported that the transcription factor nuclear factor Y binds to the murine FGFR2 proximal promoter region and activates its expression in mouse osteoblast-like cells [60].

Here we assessed the effects on FGFR2 upregulation and promoter activation of the proinflammatory cytokines that were effective in inducing epithelial cell proliferation (IL1β, IL2, IL6, TNFα and IFNγ). Among them, IL2 seemed to induce some effects on KGFR at low dose, but at higher dose it turned out to be ineffective on both KGFR and FGFR2-IIIc expression, probably due to a toxic activity. As concerning IL1β, it induced activation of FGFR2 mRNA and protein. However, no transactivation of FGFR2 promoter was observed following treatment with this cytokine, although potential elements responsive to the STAT family of transcription factors, known to be activated by interleukins [46], are present within the promoter fragment that we assayed. Therefore, it should be envisioned the possibility that IL1β activity on FGFR2 gene is mediated through elements located outside the promoter fragment that we considered in the present study. In any case, the mechanisms of FGFR2 upregulation by IL1β should be further investigated and need to be clarified.

On the other hand, TNFα induced FGFR2 expression and also showed a transcriptional activity on its promoter. Here we demonstrated that induction of FGFR2 expression by TNFα is mediated through the transcription factor E2F1. This event is consequent to hyper-phosphorylation of pRb and release of the active form of E2F1, as documented by co-immunoprecipitation and Western blot assays, rather than to an increase in total amount of E2F1 protein. Moreover, we investigated the regulation of pRb-E2F pathway by TNFα, showing that it is dependent on two different kinase families. In fact, TNFα was able to activate both p38 MAPK, which has been demonstrated to directly mediate pRb phosphorylation, and C-Raf, which has been shown to phosphorylate pRb and to be essential for its inactivation and subsequent E2F release [61].

Once released, E2F1 exerts its transcription activity on FGFR2 gene through direct binding to the promoter, as demonstrated by ChIP experiments. This finding is in keeping with similar observations reported on a rat model of vascular smooth muscle cells [47]. The hyper-phosphorylation of pRb upon FGF treatment is commonly found in cells in which these factors stimulate mitogenesis; on the contrary, in chondrocytes FGFs induce de-phosphorylation of pRb and growth arrest [62].

The observation that E2F1 transactivates FGFR2 expression led us to analyze the promoter region of the gene searching for E2F1 responding motifs. We finally were able to identify a region that lies between positions +5/+11 relative to the transcriptional initiation site, whose mutation abolished E2F1 responsiveness. This nucleotide stretch, 5′-GGCGGCG-3′, does not correspond to known E2F1 consensus motifs, neither to the non-canonical E2F binding elements previously identified on murine FGFR2 [37] or human FGFR1 [39] promoter, thus it can be considered a novel E2F1 responding box. In human FGFR1 gene, the sequences responsive to E2F1 are located close to the transcription initiation site (+4/+22 and +25/+43) [39]. This finding is in keeping with our observation in human FGFR2 gene, leading us to consider that similar mechanisms of gene activation have been conserved at least for the first two members of the FGFRs family.

Interestingly, mutation of the +47 to +53 sequence turned out in a strong FGFR2 activation by E2F1, much higher than that obtained with the wild-type construct. Such observation led us to hypothesize that this region might contain an element potentially bound by a repressor of FGFR2 transcription.

**Figure 10:**
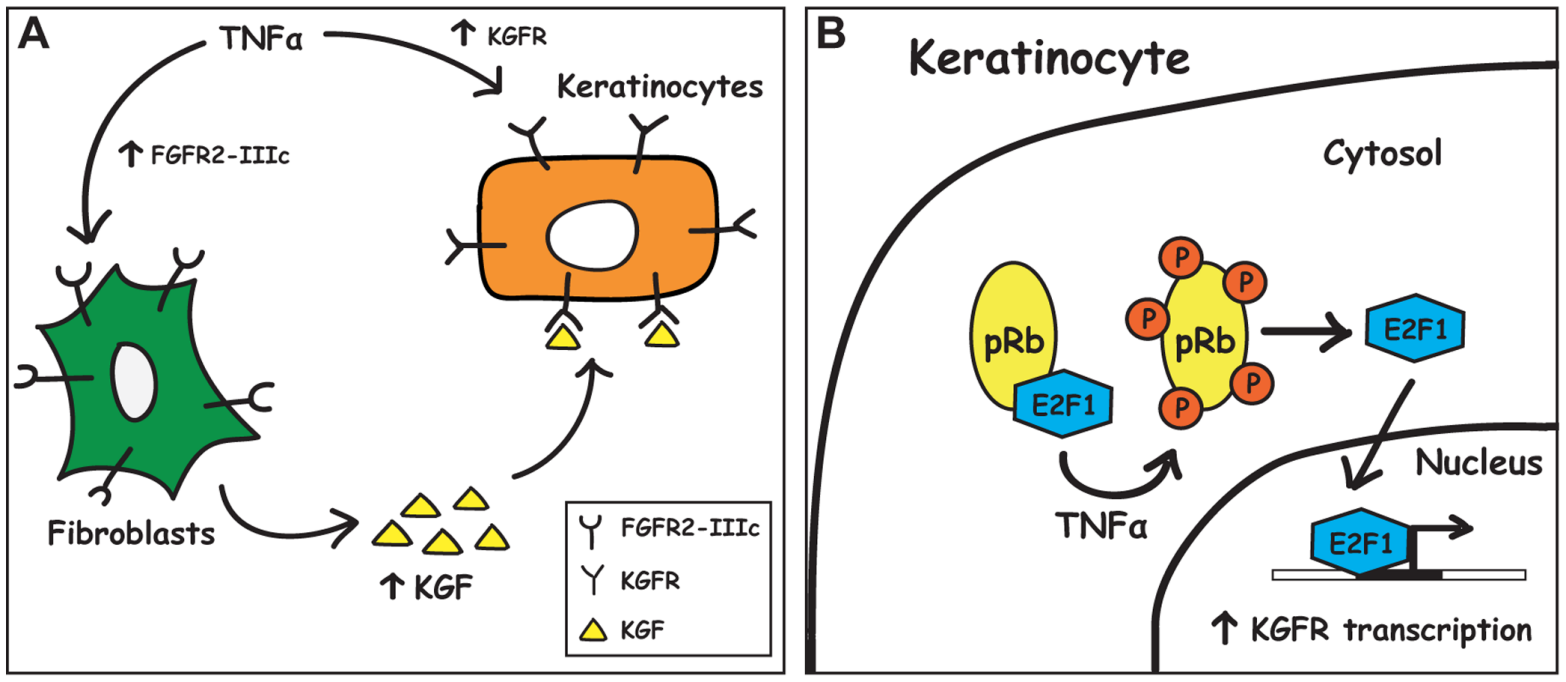
Model for TNFα effects *in vivo*. (**A**) TNFα acts on fibroblasts by stimulating an increase in FGFR2-IIIc expression and in KGF production. KGF can then bind to and activate KGFR, whose expression on keratinocytes has been at the same time stimulated by TNFα. (**B**) TNFα treatment in keratinocytes induces the hyper-phosphorylation of pRb, and the subsequent release of transcriptionally active E2F1, that can translocate from cytosol to the nucleus, where it binds FGFR2 promoter and allows for KGFR biosynthesis to occur. See text for details.

In conclusion, in the present study we suggest a simple model to explain the role and a possible mechanism of action of KGFR and TNFα in the regulation of epithelial-mesenchymal interactions during wound healing (Figure 10). The first step that occurs *in vivo* is the recruitment of TNFα to the inflammation site; then TNFα can act on both fibroblasts, by stimulating the expression of FGFR2-IIIc and the production of KGF, and keratinocytes, by inducing an increase in KGFR expression. The upregulation of both ligand (KGF) and receptor (KGFR) can stimulate KGF/KGFR signaling, with subsequent increase in epithelial cell proliferation that represents a fundamental step in re-epithelialization (Figure 10A). One of the potential mechanisms that underlie the increase of KGFR induced by TNFα involves the ability of this cytokine to phosphorylate pRb, thus allowing the release of the transcriptional factor E2F1 and its translocation from cytosol to the nucleus, where it binds FGFR2 promoter through an E2F responsive sequence within the region located at position +5/+11, driving FGFR2 gene transcription (Figure 10B).

Deregulation of FGFR2 gene has been reported in different human pathologies, such as melanoma and thyroid, breast, lung, gastric and ovarian cancers, in which up or downmodulation of KGFR expression has been observed in epithelial cells [63]–[67].

In breast cancer, this altered expression of KGF/KGFR has been extensively studied, and silencing of KGFR expression has been demonstrated to be effective in reducing cancer cells proliferation, migration and resistance to chemotherapeutic drugs [45], thus indicating that KGFR may represent an important target for the development of novel therapeutic strategies. The mechanisms that cause alterations in the expression of KGFR are still not known and it is not clear whether these events are caused by the milieu in the context of the tumor or if they directly contribute to the pathogenesis of the neoplastic disease. In any case, the modifications in KGFR expression might be related to genetic or epigenetic events affecting the promoter [68].

Thus, new evidences on the regulation of FGFR2 gene expression and a better characterization of FGFR2 promoter might turn out useful to better understand the complex network that undergoes between epithelial and mesenchymal tissues, providing both essential elements for diagnosis and prognosis of neoplastic disorders that have been associated to FGFR2 deregulation and potential targets for novel approach in tumor treatment.

## Supporting Information

Figure S1 **Effect of two doses of TNFα on KGFR protein expression in MCF-7 cells.** Western blot analysis of KGFR protein levels in MCF-7 cells untreated or treated with 50 or 100 ng/ml TNFα for 48 h. KGFR protein expression was evaluated by blotting with an anti-Bek antibody. Western blot with anti-Tubulin antibody was used as loading control. The intensity of the bands was evaluated by densitometric analysis, normalized and reported as relative expression with respect to the untreated cells.
(TIF)
