# Supplementary figures and images for: TNFα Modulates Fibroblast Growth Factor Receptor 2 Gene Expression through the pRB/E2F1 Pathway: Identification of a Non-Canonical E2F Binding Motif
Source: PLoS One. 2013 Apr 16;8(4):e61491. doi: 10.1371/journal.pone.0061491 (PMC3629046; doi:10.1371/journal.pone.0061491)

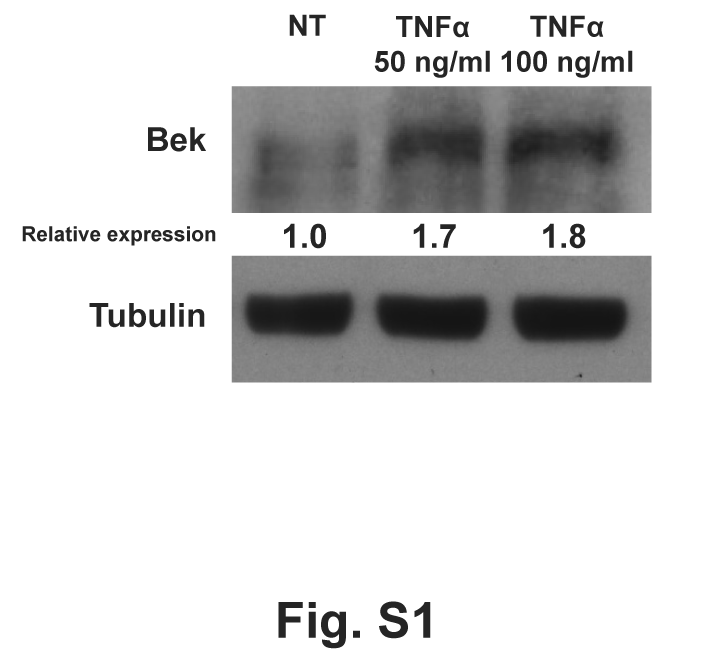

Supplement: Figure S1 — Effect of two doses of TNFα on KGFR protein expression in MCF-7 cells. Western blot analysis of KGFR protein levels in MCF-7 cells untreated or treated with 50 or 100 ng/ml TNFα for 48 h. KGFR protein expression was evaluated by blotting with an anti-Bek antibody. Western blot with anti-Tubulin antibody was used as loading control. The intensity of the bands was evaluated by densitometric analysis, normalized and reported as relative expression with respect to the untreated cells. (TIF) [file pone.0061491.s001.tif]
